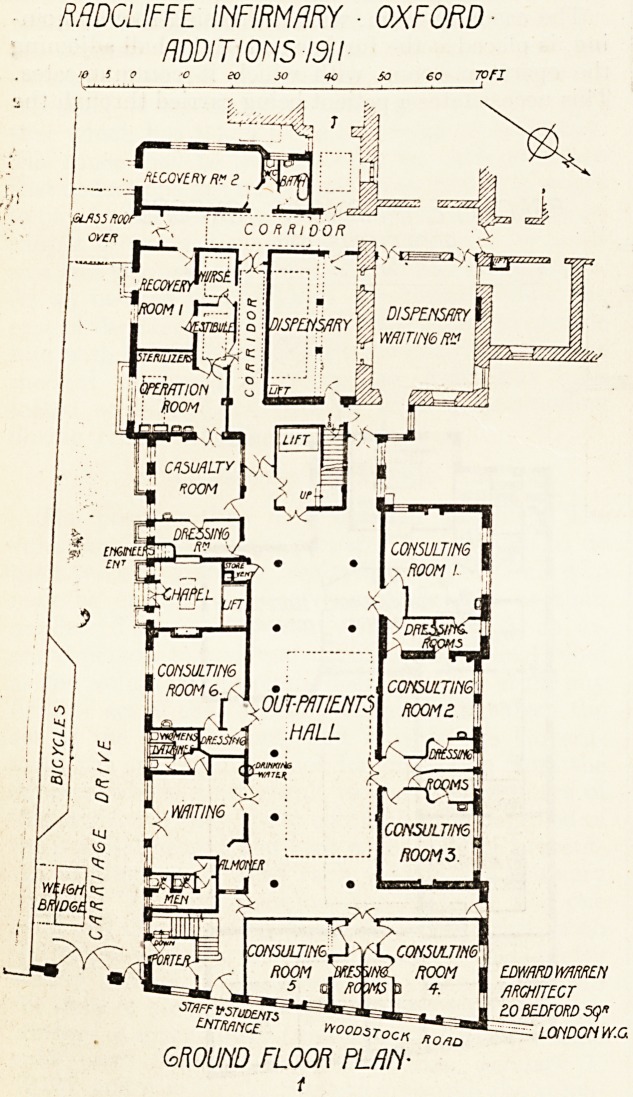# The Additions to Radcliffe Infirmary, Oxford

**Published:** 1912-02-03

**Authors:** 


### HOSPITAL ARCHITECTURE AND CONSTRUCTION.

[Communications on this subject should be marked "Architecture" in the left-hand top corner of the envelope.]

### The Additions to Radcliffe Infirmary, Oxford.

The new wing which is about to be built at this hospital is to take the place of the old one-story out-patient department, which projects from the main building at its north-east corner, and the old mortuary building, which occupies the north-east corner of the site. The north-east front of the new building will therefore be hard on the boundary in Woodstock Road and the north-west front will face the entrance courtyard; on the south-east the building will be separated from the boundary wall and buildings of Somerville College by a narrow roadway, while the south-west end is joined up to the old hospital.

The entrance for out-patients is in the carriage drive on the north-east side; a small waiting-hall, off which the patients' latrines are arranged, communicates with the almoner's office and with the main waiting-hall, a long and somewhat narrow room lighted by a large skylight. The six consulting-rooms, with their six dressing-rooms, are ranged round three sides of this hall.

The entrance doors to the consulting-rooms open not direct into the hall but into lobbies, and each of the dressing-rooms, except in one instance, has a door of exit to the lobby. Communication between the three consulting-rooms on the north-east side is arranged without passing through the hall. There is no indication on the plan of how these lobbies are lighted, but they must infallibly be dark unless it be intended to provide roof-lights.

The casualty-room, with a dressing-room adjoining, is placed at the further end of the hall adjoining the operation-room, with which it communicates. This necessitates a patient being carried through the greater part of the hall to reach the casualty-room. Surely it would have been possible to have arranged for a separate entrance to the casualty-room from the outside; a bad accident could then have been driven up to the door, and the necessity for carrying the patient through the hall before the waiting patients avoided. Between the casualty dressing-room and consulting-room No. 6 is the mortuary chapel, which is entirely shut off from the out-patient department. A lift communicates with the mortuary below and the pathological department above.

The 1911 Additions in Progress at Radcliffe Infirmary.

The operation-room, with a nurses' room and two large recovery-rooms, adjoins the casualty-room. At the south-west end of the hall is the staircase to the upper floors, with a lift for taking up helpless patients. Beyond this is the dispensary, with the medicine waiting-room adjoining. This last is a room in the old building now used as dispensary. The patients' exit is in the same carriage road as their entrance.

The first floor contains a complete electric department, two rooms for dental surgery, with small

RADCLIFFE INFIRMARY · OXFORD ·
ADDITIONS 1911

GROUND FLOOR PLAN·

workshop for mechanical work, and a vaccine-room. In the front is a staff-room and lavatory and living and bedrooms for porters. The second floor contains rooms for the teaching staff, laboratories for chemistry and bacteriology, post-mortem room with pathological laboratory, and a lecture-room for nurses. The basement is to contain store-cellars, chambers for calorifiers and refrigerators, the mortuary already referred to, and a store for bicycles.

The buildings have been designed by Mr. Edward Warren, F.R.I.B.A., in collaboration with Dr. W. J. Mackintosh, M.B., M.V.O., Superintendent of the Western Infirmary, Glasgow.

## Figures and Tables

**Figure f1:**
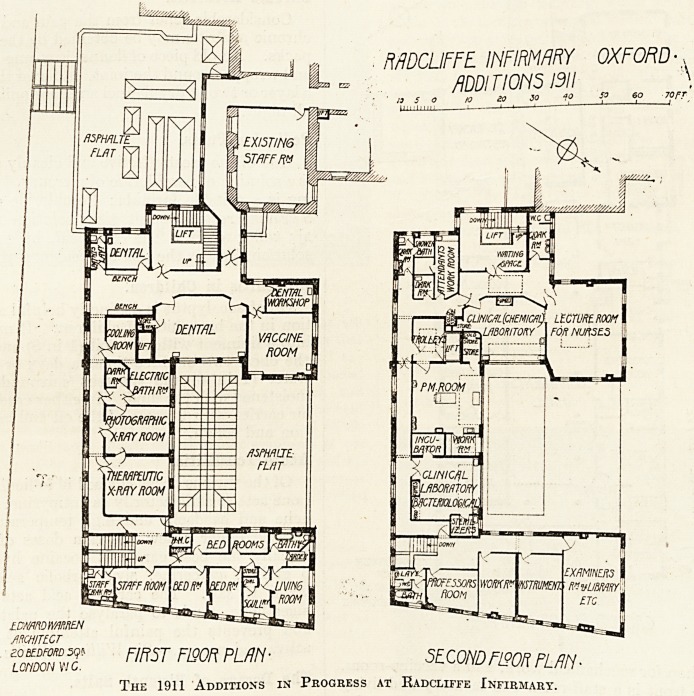


**Figure f2:**